# Urinary Dickkopf-related protein 3 as a novel biomarker for kidney function decline in children with Alport syndrome

**DOI:** 10.1007/s00467-025-06696-3

**Published:** 2025-02-04

**Authors:** Jan Boeckhaus, Burkhard Tönshoff, Lutz T. Weber, Lars Pape, Kay Latta, Henry Fehrenbach, Baerbel Lange-Sperandio, Matthias Kettwig, Sabine König, Ulrike John-Kroegel, Jutta Gellermann, Matthias Galiano, Sima Jami, Dennis Pieper, Gry Helene Dihazi, Angelika Hafke, Stefan Kohl, Max C. Liebau, Jens König, Dieter Haffner, Oliver Gross, Manuel Wallbach

**Affiliations:** 1 Clinic for Nephrology and Rheumatology, University Medical Center Göttingen, Goettingen, Germany; 2 Department of Pediatrics I, University Children’s Hospital Heidelberg, Heidelberg, Germany; 3 Pediatric Nephrology, Children’s and Adolescents’ Hospital, University of Cologne, Faculty of Medicine and University Hospital Cologne, Cologne, Germany; 4 Department of Pediatric Kidney, Liver and Metabolic Diseases, Hannover Medical School, Hannover, Germany; 5 Department of Pediatrics II, University Children’s Hospital, University of Duisburg-Essen, Essen, Germany; 6 Clementine Kinderhospital Frankfurt, Frankfurt, Germany; 7 Pediatric Nephrology, Children’s Hospital, Memmingen, Germany; 8 Dr. V, Hauner Children’s Hospital, Ludwig Maximilian University, Munich, Germany; 9 Department of Pediatrics and Adolescent Medicine, University Medical Center Göttingen, Göttingen, Germany; 10 University Children’s Hospital Münster, Münster, Germany; 11 Division of Pediatric Nephrology, University Children’s Hospital, Jena, Germany; 12 Pediatric Nephrology, Charité Children’s Hospital, Berlin, Germany; 13 Department of Pediatrics and Adolescent Medicine, University Hospital, Friedrich-Alexander-University Erlangen, Erlangen, Germany; 14 Department of Clinical Chemistry, University Medical Center Göttingen, Göttingen, Germany; 15 Department of Pediatrics, Center for Family Health, Center for Molecular Medicine and Center for Rare Diseases, University Hospital Cologne and Faculty of Medicine, University of Cologne, Cologne, Germany; 16 German Center for Cardiovascular Research (DZHK), Partner Site Göttingen, Göttingen, Germany

**Keywords:** Hereditary kidney disease, Alport syndrome, Dickkopf-related protein 3, DKK3, Albuminuria, Kidney failure

## Abstract

**Background:**

Chronic kidney disease (CKD) seriously affects the well-being and shortens the life expectancy of children and adolescents, but its progression is challenging to predict. Therefore, there is an urgent need for biomarkers that can identify children at risk of faster CKD progression. Alport syndrome (AS) is the most common monogenetic glomerular kidney disease. Urinary Dickkopf-related protein 3** (**DKK3) is associated with a decline in estimated glomerular filtration rate (eGFR) in adults and children with advanced CKD. However, its potential for early detection of CKD and its prognostic value in children with AS remain unknown.

**Methods:**

Urine samples from 49 children enrolled in the EARLY PRO-TECT Alport trial were analyzed to evaluate whether DKK3 could identify children with AS to be at risk for faster CKD progression.

**Results:**

DKK3 levels in patients with AS were higher than those of healthy individuals reported in the literature. DKK3 levels were more elevated in patients with later stages of AS. Furthermore, children who were not treated with renin angiotensin system inhibitors (RASi) had higher DKK3 levels than treated children. Children with above-average DKK3 levels were more likely to have increased albuminuria after 2 years of follow-up than children with below-average DKK3 levels.

**Conclusion:**

Urinary DKK3 is significantly elevated in children at early stages of AS. There was a potential association between higher DKK3 levels, worsening albuminuria, and a decline in kidney function. These findings suggest that DKK3 may be a prognostic marker for predicting the risk of kidney damage in children with AS.

**Graphical Abstract:**

**Figure:**
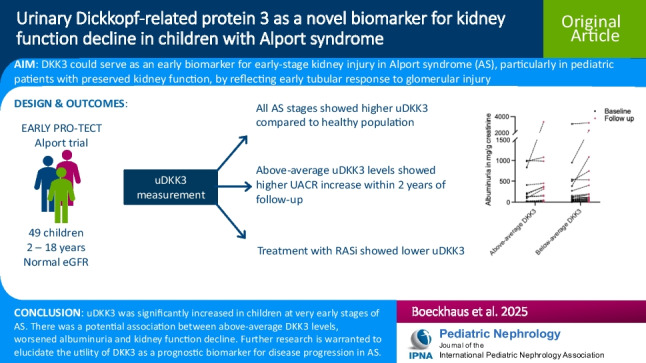
A higher-resolution version of the Graphical abstract is available as Supplementary information

**Supplementary Information:**

The online version contains supplementary material available at 10.1007/s00467-025-06696-3.

## Background

Chronic kidney disease (CKD) seriously impacts the well-being and shortens the life expectancy of children and adolescents, but its progression is challenging to predict [1, 2]. Therefore, there is an urgent need for biomarkers that can identify children at risk of faster CKD progression. The most frequent underlying causes of CKD in children are congenital anomalies of the kidney and urinary tract and monogenic kidney disease [3]. Alport syndrome (AS) is the most common monogenetic glomerular kidney disease [4, 5]. AS is a disease of type IV collagen, which is an essential component of the glomerular basement membrane in the kidney, eyes, and cochlea [6]. The disruption of type IV collagen structure in AS results in impaired function of the glomerular basement membrane filtration barrier, leading to increased albuminuria, which may further promote disease progression [7, 8]. Consequently, patients with AS have a very high risk for progression to kidney failure (KF), which is frequently accompanied by progressive sensorineural hearing loss and ocular lesions [7, 9–11]. While most patients with AS inherit the disease through an X-linked pattern caused by pathogenic variants in the *COL4A5* gene, up to 30% of patients are diagnosed with autosomal recessive or dominant AS caused by pathogenic variants in both alleles of *COL4A3* or *COL4A4* genes [12–14].

Heterozygous patients with autosomal AS and female patients with heterozygous X-linked AS have a wide range of clinical outcomes [15, 16]. Genetic testing of *COL4A* genes has emerged as the gold standard for the diagnosis of AS, and due to the complexity of genetic testing in AS, a close collaboration between clinicians and geneticists is recommended [17, 18]. Registry data have demonstrated that inhibition of the renin-angiotensin system (RASi) by angiotensin receptor blockade (ARB) or angiotensin-converting enzyme inhibitors (ACEi) can delay kidney failure in patients living with AS by years. However, many patients with AS still reach KF at a relatively young age [19]. To investigate whether initiating treatment in early stages of AS is beneficial, the EARLY PRO-TECT Alport trial (NCT01485978) was conducted. Early ramipril treatment revealed no safety concerns compared with placebo and showed a trend toward reduced disease progression [20]. The fast progression of kidney disease in some patients as well as the substantial variation in clinical outcomes in other patients necessitates the development of predictors for faster decline of kidney function. Upon acute or sustained stress acting on the tubular compartment, Dickkopf-related protein 3 (DKK3) is released by the kidney tubular epithelial cells into the urine [21, 22]. Higher urinary DKK3 is associated with a pronounced decline of estimated glomerular filtration rate (eGFR) in adult patients and children with advanced CKD [3, 23]. DKK3’s potential for early detection of CKD in children with AS and its prognostic value on kidney function remain unknown. Therefore, the aim of this study was to investigate whether DKK3 can be detected in the urine of pediatric patients with early stages of AS and to evaluate its association with kidney failure. We hypothesized that DKK3 could serve as an early biomarker for early-stage kidney injury in AS, particularly in pediatric patients with preserved kidney function, by reflecting early tubular response to glomerular injury. Urine samples from children enrolled in the EARLY PRO-TECT Alport trial were analyzed to address this hypothesis.

## Methods

### Clinical data and sampling of urine

Clinical and demographic data were collected as part of the EARLY PRO-TECT Alport trial; the patient cohort has been reported previously [10, 18, 20, 24, 25]. The study enrolled children with a confirmed diagnosis of AS, aged between 2 and 18 years, and possessing normal glomerular filtration rates. The trial design was randomized, placebo-controlled, and double-blind. The study was registered on ClinicalTrials.gov (NCT01485978). Twenty children were randomly assigned to the intervention group, while the remaining 42 formed an open-label control group. The treatment phase lasted for 3 to 6 years. Institutional review board approval was obtained from the Ethics Committee of the University Medical Center Göttingen (AZ 11/06/11) and of each institution involved with the EARLY PRO-TECT Alport trial. The study was performed in accordance with the ethical standards as laid down in the 1964 Declaration of Helsinki and its later amendments.

Written informed consent was obtained from all legal guardians, and assent was obtained from all participants 6 years of age or older. During the trial, urine samples were collected every 6 months and albuminuria was measured to monitor disease progression during the trial [26]. eGFR was estimated using the Schwartz formula.

### Intervention and outcome measures

Stages of AS were defined as stage 0: albuminuria < 30 mg/g creatinine, stage I: albuminuria: 30–300 mg albumin/g creatinine, and stage II: albuminuria: > 300 mg/g creatinine [26].

To investigate the influence of genotype, patients with XLAS were divided into two groups according to the pathogenic variants detected in *COL4A5*. Splicing variants, duplications, and deletions were defined as severe variants, whereas missense variants were defined as less severe variants [26].

### Assessment of DKK3

In this analysis, the commercially available ELISA test kit (ReFiNE, DiaRen, Homburg/Saar, Germany) was used for analysis with the associated implementation protocol supplied by the manufacturer.

### Statistical methods

Statistical comparisons were not formally powered or prespecified. Continuous variables were presented as mean and standard deviation (SD) or as median and inter-quartile range (IQR) and categorical variables as percentages. Comparison of means between groups was conducted with the unpaired Student’s t-test in the case of roughly normally distributed values. If normality was not assumed, the Mann–Whitney test or Kruskal–Wallis test was used to compare groups. The Sign test was used for intra-individual comparison if normality was not assumed and a paired t-test was used in case of roughly normally distributed values.

Spearman’s correlation was performed to assess correlations. Probability values (*p-*values) below 0.05 were considered statistically significant. Data analysis was performed using IBM SPSS Statistics (version 28 for MacOS, IBM Corporation, Armonk, NY, USA). GraphPad Prism (version 10 for MacOs, GraphPad Software, San Diego, California, USA) was used to generate figures.

## Results

### Demographic and clinical characteristics

Table 1 shows characteristics from the 49 children included in this study at baseline. The mean age at baseline was 9.1 ± 4.3 years. Forty-eight children were male (98%). The mode of inheritance was X-linked in 43 children (88%), autosomal in five children (10%; autosomal recessive in four children and autosomal dominant in one child), and unknown in one child (2%). Children were in early stages of AS with a mean eGFR of 127 ± 25 ml/min/1.73 m^2^ and a median albuminuria of 60 mg/gram creatinine (mg/g creatinine) (IQR 169). The mean intra-patient standard deviation of eGFR measurements within 6 months was 16.8 ± 8.6 ml/min/1.73 m^2^ (447 measurements). Most children were in AS stage 0 (43%). The mean BMI was 17.9 ± 3.8 kg/m^2^, and mean blood pressure was 105 ± 13/60 ± 9 mmHg. At baseline, 41 children were treated with RASi (84%).

**Table 1:** Demographic and clinical characteristics of 49 children examined in this trial at measurement of DKK3 (baseline). *BMI*, body mass index; *RASi*, inhibitors of the renin-angiotensin system. Values are mean + SD, median (IQR), or *n* (%) as appropriate

	*N*	
Age (years)	49	9.1 ± 4.3
Male no. (%)		48 (98)
Mode of inheritance (%)		
X-linked		43 (88)
Autosomal		5 (10)
Unknown		1 (2)
Creatinine (mg/dl)		0.5 ± 0.2
eGFR (ml/min/1.73 m^2^)		127 ± 25
Albuminuria (mg/g creatinine)		60 (169)
AS stage		
0		21 (43)
I		19 (39)
II		9 (18)
BMI (kg/m^2^)		17.9 ± 3.8
Systolic/diastolic blood pressure (mmHg)		105 ± 13/60 ± 9
RASi		41 (84)

### DKK3 levels at baseline

DKK3 levels were measured in urine and normalized to urine creatinine. The median DKK3 was 151 pg/mg creatinine (IQR 180), while the mean DKK3 was higher (257 ± 293 pg/mg creatinine).

**Fig. 1:**
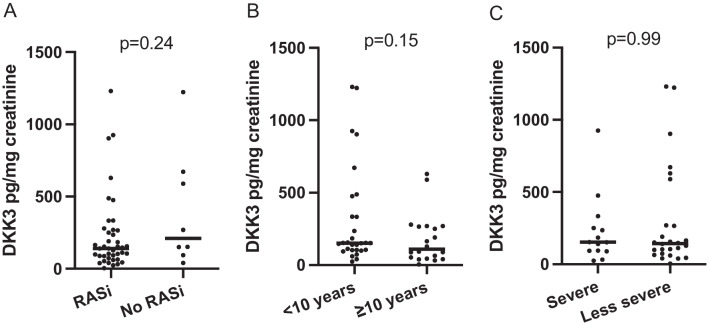
**A** Urinary DKK3 in pg/mg creatinine in comparison of children treated with RASi (RASi) and children without RASi (No RASi) at baseline. **B** Urinary DKK3 in pg/mg creatinine in comparison of children < 10 years and children ≥10 years old at baseline. **C** Urinary DKK3 in pg/mg creatinine in comparison of children with severe and less severe variants at baseline

**Fig. 2:**
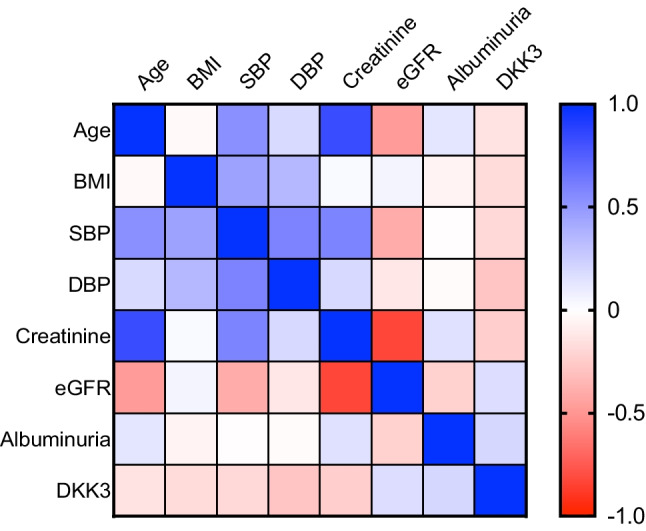
Spearman ρ at baseline. BMI, body-mass-index percentile; SBP, systolic blood pressure; DBP, diastolic blood pressure; eGFR, estimated glomerular filtration rate in ml/min/1.73 m^2^; Albuminuria, albuminuria in mg/g creatinine; DKK3, urinary Dickkopf-related-protein 3 in pg/mg creatinine (*n* = 49)

Median DKK3 was 112 pg/mg creatinine (IQR 91) in children with AS stage 0 (*n* = 21), 153 pg/mg creatinine (IQR 314) in children with AS stage I (*n* = 19) (*p* = 0.25), and 191 pg/mg creatinine (IQR 246) in children with AS stage II (*n* = 9) (*p* = 0.39) (Table 2). The 8 children who were not treated with RASi at baseline had a median albuminuria of 210 mg/g creatinine (IQR: 473), while the 41 children treated with RASi had a lower median DKK3 level of 141 pg/mg creatinine (IQR 177) (*p* = 0.24) (Fig. 1). A median DKK3 level of 152 pg/mg creatinine (IQR 230) was observed in children younger than 10 years (mean age 5.9 years; n = 29), compared to 111 pg/mg creatinine (IQR: 215) in those 10 years or older (mean age 13.7 years; *n* = 20) (*p* = 0.15) (Fig. 1). The correlation coefficient between age and urinary DKK3 at baseline was − 0.14 (*p* = 0.34). At baseline, the correlation coefficient between albuminuria and urinary DKK3 was 0.2 (*p* = 0.17) (Fig. 2).

**Table 2:** Alport syndrome stage, amount of albuminuria in mg/g creatinine and DKK3 levels in pg/mg creatinine at measurement of DKK3. *AS stage 0*, albuminuria < 30 mg/g creatinine; *AS stage I*, albuminuria 30–300 mg/g creatinine; *AS stage II*, albuminuria > 300 mg/g creatinine. Values are mean + SD or median (IQR) as appropriate. *Mann–Whitney test (AS stage 0 vs. I and AS stage 0 vs. II)

AS stage	*N*	Albuminuria (mg/g creatinine)	DKK3 level (pg/mg creatinine)	p*
0	21	14 ± 7	112 (91)	
I	19	117 ± 69	153 (314)	0.25
II	9	965 ± 840	191 (246)	0.39

### Genotype-phenotype correlation

For genotype–phenotype correlation, the 42 male children with X-linked AS were divided into two groups depending on their pathogenic variant causing AS. Severity of the variant was unclear in two children. Out of 40 children, 14 (35%) had severe variants and 26 (65%) children had less severe variants. At baseline, children with less severe variants were older (8.8 ± 4.2 vs. 7.9 ± 3.9 years; *p* = 0.49), had a higher eGFR (132 ± 22 vs.124 ± 27 ml/min/1.73 m^2^; *p* = 0.32), and a significantly lower median amount of albuminuria (21, IQR 54 vs. 127, IQR 155 mg/g creatinine; *p* = 0.007). There was no difference in the median DKK3 level in children with severe variants (153 pg/mg creatinine (IQR 154)) compared to children with less severe variants (144 pg/mg creatinine (IQR 188)) (*p* = 0.99).

### DKK-3 as a potential biomarker of disease progression

**Fig. 3:**
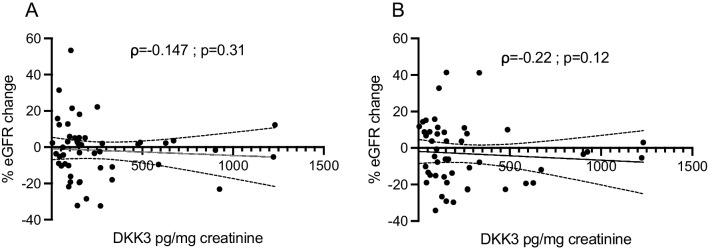
**A** Correlation of urinary DKK3 in pg/mg creatinine and % eGFR change from baseline after a half-year follow-up (*n* = 49). **B** Correlation of urinary DKK3 in pg/mg creatinine and % eGFR change from baseline after a 2-year follow-up (*n* = 49)

**Fig. 4:**
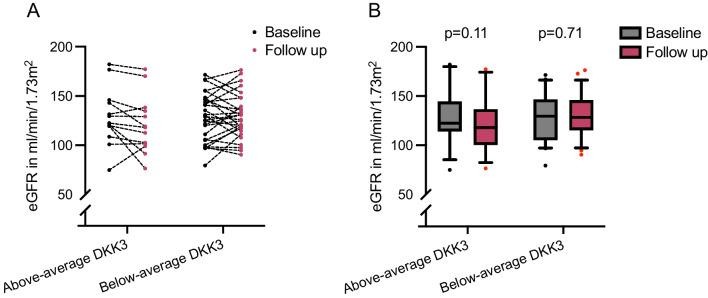
**A** Intraindividual change of estimated GFR in ml/min/1.73 m^2^ from baseline to follow-up after 1 year separated by mean DKK3 levels (*n* = 41) **B** Group comparison of estimated GFR in ml/min/1.73 m^2^ in children with above- and below-average DKK levels from baseline to follow-up after 1 year (*n* = 41); whiskers indicate 10–90 percentile; *p*-values indicate the significance of differences within groups

A correlation of DKK3 and change of albuminuria or eGFR was unstudied in AS. Therefore, this correlation was assessed at different timepoints. The correlation coefficient between baseline DKK3 levels and the percentage change of GFR after a half-year follow-up was ρ = − 0.147 (*p* = 0.31) and ρ = − 0.22 two years after baseline (*p* = 0.12) (*n* = 49; Fig. 3). The highest correlation between baseline urinary DKK3 and the intraindividual change of albuminuria from baseline to follow-up was observed after 2 years of follow-up (ρ = 0.27; *p* = 0.087; *n* = 41). In 41 children, data on eGFR and albuminuria 2 years after baseline were available. The mean urinary DKK3 level in these 41 children was 257 ± 294 pg/mg creatinine (median DKK3 level 151 pg/mg creatinine (IQR 180)). At baseline, children with above-average DKK3 levels (DKK3 > 257 pg/mg creatinine; *n* = 13) and children with below-average DKK3 levels (*n* = 28) were similar in age (9.4 ± 5.2 vs. 9.1 ± 3.9 years) and had similar eGFR (129 ± 29 vs. 128 ± 25 ml/min/1.73 m^2^). The above-average-DKK3 group had numerically higher albuminuria at baseline (136 mg/g creatinine (IQR 380) vs. 54 mg/g creatinine (IQR 132); *p* = 0.1). Four children in the above-average DKK3 group (31%) and four children (14%) in the below-average DKK3 group were not treated with RASi. In children in the above-average DKK3 group, eGFR decreased by − 6 ml/min/1.73 m^2^ (IQR 11), while it remained stable in the below-average DKK3 group (1 ml/min/1.73 m^2^ (IQR 22)) after 1-year follow-up (*p* = 0.10) (Fig. 4). Nine of 13 children (69%) with above-average urinary DKK3 had higher albuminuria 2 years after baseline. In three children (23%), albuminuria remained stable (± 10 mg/g creatinine) and decreased in one child (8%). In contrast, in the 28 children with urinary DKK3 concentration below average, albuminuria increased in 13 children (46%) and remained stable in 10 children (36%) but decreased in five children (18%).

**Fig. 5:**
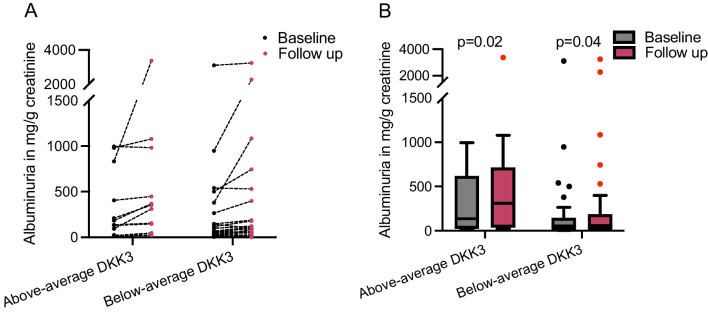
**A** Intraindividual change of albuminuria in mg/g creatinine from baseline to follow-up after 2 years separated by mean DKK3 levels (*n* = 41). **B** Turkey box plots of albuminuria in mg/g creatinine in children with above- and below-average DKK3 levels at baseline and after a 2-year follow-up (*n* = 41); *p*-values indicate the significance of differences within groups

The median albuminuria increased from 136 mg/g creatinine (IQR 380) to 310 mg/g creatinine (IQR 401) in children with above-average DKK3 levels (*p* = 0.02), while the median albuminuria remained stable in children with below-average DKK3 levels (from 54 mg/g creatinine (IQR 132) to 59 mg/g creatinine (IQR 164)) (*p* = 0.04) (Fig. 5).

## Discussion

This study investigated the novel urinary biomarker DKK3 and showed that DKK3 levels were elevated in very early stages of CKD in children with AS. DKK3 levels in this study were substantially higher than those reported in adults (I LIKE HOMe Study 33 pg/mg creatinine) and children (11 pg/mg creatinine) without CKD in previous studies [23, 27]. DKK3 levels were numerically higher in children with later AS stages (112 pg/mg creatinine (IQR 91) in children with AS stage 0 vs. 191 pg/mg creatinine (IQR 246) in children with AS stage II). Interestingly, children who were not treated with RASi had higher DKK3 levels compared to children who were treated with RASi (210 pg/mg creatinine (IQR 473) vs. 141 pg/mg creatinine (IQR 177); *p* = 0.24*).* The renin-angiotensin-system and the Wnt/β-catenin pathway were reported to be interconnected, with RAS activation potentially influencing DKK3 expression, as angiotensin II, a key RAS component, can activate the Wnt/β-catenin pathway via the pro-renin receptor [28–31]. Further research is warranted to investigate how the Wnt/β-catenin pathway is involved in the pathogenesis of AS. This observation is consistent with findings of samples used from the 4C study, which showed that RASi lowered urinary DKK3 levels [3].

Previous research investigated urinary DKK3 levels in samples from the ESCAPE trial (a European randomized controlled trial with 468 children aged 3–18 years with an eGFR of 15–80 ml/min/1.73 m^2^) and the 4C study (a European observational study with 704 children aged 6–17 with an eGFR of 10–60 ml/min/1.73 m^2^) [3]. These studies suggested that DKK3 levels could be a valuable tool to assess the effects of treatments and identify children at high risk for worsening kidney function [3]. DKK3 emerges as a promising biomarker for CKD progression by reflecting tubular injury [3, 21, 22, 32–36]. Tubulointerstitial fibrosis, a hallmark of CKD, is driven by dysfunctional tubular epithelial cells (TECs) that produce DKK3 [37–39]. This protein influences the Wnt/β-catenin pathway, crucial for kidney cell function [40, 41]. In AS, the disruption of the GBM causes hematuria, proteinuria, and progressive focal segmental glomerulosclerosis of the glomeruli. Leakage of proteins into the tubular lumen damages TECs and triggers tubulointerstitial fibrosis [6, 7, 42]. Consequently, elevated DKK3 levels might reflect the severity of tubular stress. This link between DKK3 and tubular injury in AS suggests its potential as a potential prognostic biomarker for disease progression. This study focused on children with AS in very early stages of the disease. Interestingly, even in these earlier stages of CKD compared to the ESCAPE and 4C studies, significantly elevated DKK3 levels were found. In this study, 69% of children with above-average DKK3 levels had increased albuminuria after 2 years, compared to 46% of children with below-average levels. Additionally, the rise in albuminuria was steeper in the high DKK3 group. Since albuminuria can be considered as a predictor of worsening kidney function [43], these findings suggest that DKK3 could be a prognostic marker in children with AS, helping to predict disease progression. In this study, no statistically significant correlation was found between DKK3 and the change in eGFR. One possible explanation could be the early stage of kidney failure in the children of this study (baseline eGFR 127 ± 25 ml/min/1.73 m^2^). GFR is highly variable in this range, which may have influenced the results. For example, the mean intra-patient standard deviation of eGFR measurements within 6 months in this study was 16.8 ± 8.5 ml/min/1.73 m^2^.

Moving forward, it seems reasonable to investigate DKK3 in larger studies at very early stages of various childhood CKD. This will help to determine whether DKK3 can be used as a reliable early marker to identify children at risk of developing chronic kidney disease. A strength of this study is the clinically well-characterized cohort due to its use of data from a randomized controlled trial. However, limitations exist. The relatively small number of children enrolled may have masked any significant correlations. Additionally, DKK3 measurements were not available for all participants of the EARLY PRO-TECT Alport trial, and complete follow-up data regarding albuminuria were not available for the entire cohort. Furthermore, the study analyzed samples from primarily White boys, limiting the generalizability of the findings to other ethnicities and girls. Therefore, a cautious interpretation of the results is warranted due to these limitations. Future studies will be necessary to confirm urinary DKK3 as a prognostic marker in AS, which might enable the concept of personalized treatment strategies in children and adults with AS with the growing number of potential treatment options [44, 45].

## Conclusion

This study demonstrated that urinary DKK3, a biomarker for CKD progression by reflecting tubular injury, was significantly increased in children at very early stages of AS. DKK3 was numerically lower in children treated with RASi compared to those without RASi therapy. While correlations between DKK3 levels and baseline clinical parameters were modest, 2-year follow-up data indicated a potential association between above-average DKK3 levels, worsened albuminuria, and kidney function decline. Further research is warranted to elucidate the utility of DKK3 as a prognostic biomarker for disease progression in AS.

## Supplementary Information

Below is the link to the electronic supplementary material.

Graphical abstract (PPTX 112 KB)

## Data Availability

Data supporting reported results can be requested from the corresponding author on reasonable request.
