# Supplementary material for: Urinary Dickkopf-related protein 3 as a novel biomarker for kidney function decline in children with Alport syndrome
Source: Pediatr Nephrol. 2025 Feb 4;40(7):2205–13. doi: 10.1007/s00467-025-06696-3 (PMC12116715; doi:10.1007/s00467-025-06696-3)
Supplement: Supplementary file 1 — Graphical abstract (PPTX 112 KB) [file 467_2025_6696_MOESM1_ESM.pptx]

## Slide 1
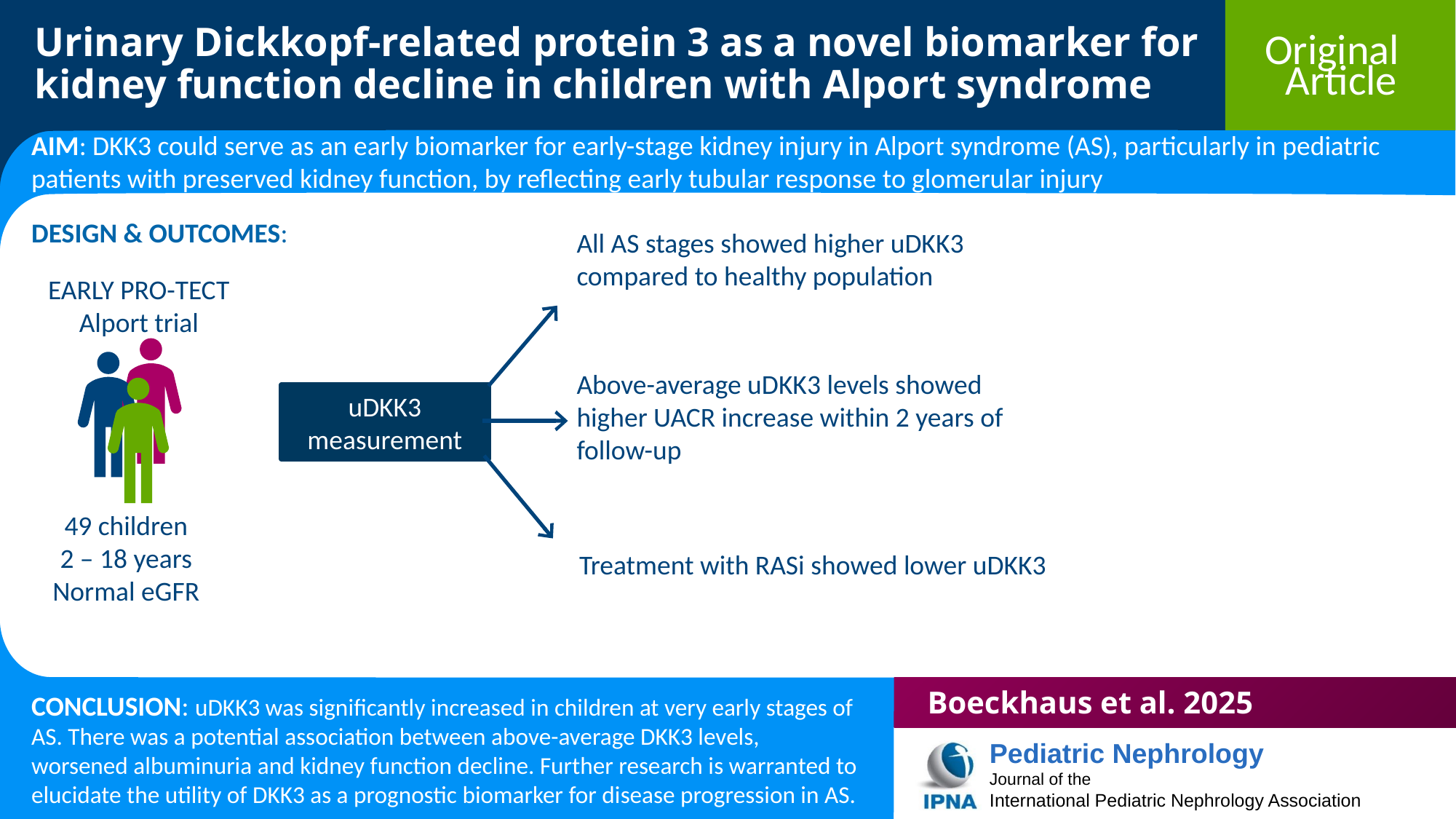

Urinary Dickkopf-related protein 3 as a novel biomarker for kidney function decline in children with Alport syndrome
AIM: DKK3 could serve as an early biomarker for early-stage kidney injury in Alport syndrome (AS), particularly in pediatric patients with preserved kidney function, by reflecting early tubular response to glomerular injury
DESIGN & OUTCOMES:
All AS stages showed higher uDKK3 compared to healthy population
EARLY PRO-TECT Alport trial
Above-average uDKK3 levels showed higher UACR increase within 2 years of follow-up
uDKK3 measurement
49 children
2 – 18 years
Normal eGFR
Treatment with RASi showed lower uDKK3
Boeckhaus et al. 2025
CONCLUSION: uDKK3 was significantly increased in children at very early stages of AS. There was a potential association between above-average DKK3 levels, worsened albuminuria and kidney function decline. Further research is warranted to elucidate the utility of DKK3 as a prognostic biomarker for disease progression in AS.
